# Antiparasitic activity of chicory (*Cichorium intybus*) and its natural bioactive compounds in livestock: a review

**DOI:** 10.1186/s13071-018-3012-4

**Published:** 2018-08-22

**Authors:** Miguel Peña-Espinoza, Angela H. Valente, Stig M. Thamsborg, Henrik T. Simonsen, Ulrik Boas, Heidi L. Enemark, Rodrigo López-Muñoz, Andrew R. Williams

**Affiliations:** 1 Instituto de Farmacologia y Morfofisiologia, Facultad de Ciencias Veterinarias, Universidad Austral de Chile, Valdivia, Chile; 2 Department of Veterinary and Animal Sciences, Faculty of Health and Medical Sciences, University of Copenhagen, Dyrlægevej 100, 1870 Frederiksberg C, Denmark; 3 Department of Biotechnology and Biomedicine, Technical University of Denmark, Søltofts Plads, 2800 Kongens Lyngby, Denmark; 4 National Veterinary Institute, Technical University of Denmark, Kemitorvet, 2800 Kongens Lyngby, Denmark; 5 Norwegian Veterinary Institute, Ullevålsveien 68, P.O. Box 750, N-0106 Oslo, Sentrum Norway

**Keywords:** Chicory, Antiparasitic, Helminths, Protozoa, Livestock, Bioactive compounds, Sesquiterpene lactones, Nutraceutical

## Abstract

Increasing drug resistance in gastrointestinal (GI) parasites of livestock and concerns about chemical residues in animal products and the environment are driving the development of alternative control strategies that are less reliant on the use of synthetic drugs. An increasingly investigated approach is the use of bioactive forages with antiparasitic properties as part of the animal’s diet (nutraceuticals) or as potential sources of novel, natural parasiticides. Chicory (*Cichorium intybus*) is a multi-purpose crop and one of the most promising bioactive forages in temperate regions, and numerous *in vivo* trials have explored its potential against parasitic nematodes in livestock. However, it is unclear whether chicory can induce a direct and broad activity against various GI parasites in different livestock species, and the levels of chicory in the diet that are required to exert an efficient antiparasitic effect. Moreover, the mechanisms leading to the reported parasiticidal activity of chicory are still largely unknown, and its bioactive phytochemicals have only recently been investigated. In this review, we summarise the progress in the study of the antiparasitic activity of chicory and its natural bioactive compounds against GI parasites in livestock, through examination of the published literature. The available evidence indicates that feeding chicory can reduce faecal egg counts and/or worm burdens of abomasal nematodes, but not infections with intestinal worms, in ruminants. Highly chicory-rich diets (≥ 70% of chicory dry matter in the diet) may be necessary to directly affect abomasal parasitism. Chicory is known to synthesise several bioactive compounds with potential antiparasitic activity, but most research has been devoted to the role of sesquiterpene lactones (SL). Recent *in vitro* studies have confirmed direct and potent activity of SL-rich extracts from chicory against different GI helminths of livestock. Chicory SL have also been reported to exhibit antimalarial properties and its potential antiprotozoal activity in livestock remains to be evaluated. Furthermore, the detailed identification of the main antiparasitic metabolites of chicory and their pharmacokinetics need further confirmation. Research gaps and perspectives on the potential use of chicory as a nutraceutical forage and a source of bioactive compounds for parasite control in livestock are discussed.

## Background

Infections with gastrointestinal (GI) parasites are ubiquitous in grazing livestock worldwide, inducing subclinical and clinical diseases that can markedly impair animal health, food production and agricultural economies [1–3]. In ruminants, abomasal and intestinal nematodes are among the most pathogenic GI parasites and efficient deworming programs to control them are essential for sustainable animal farming [4, 5]. Currently, nematode control in livestock largely relies on repeated treatments with synthetic anthelmintic drugs. However, the sustainability of this approach is at risk due to the rapid spread of drug-resistant parasite populations [6–8]. Furthermore, there is a growing public awareness, in several countries, of the need to reduce the prophylactic use of veterinary drugs and the subsequent risk of chemical residues in animal products and the environment [9–11]. Outdoor-reared and pasture-based livestock are also progressively being promoted to enhance grassland biodiversity and avoid the feeding of animals with human-edible crops [12–14], but an inevitable consequence of these production systems is the persistent exposure to parasitic infections. Consequently, it is imperative to generate novel antiparasitic tools to reduce the dependence on existing drugs and progress towards the development of integrated and sustainable parasite control strategies [15, 16].

An increasingly explored approach is the use of bioactive forages with antiparasitic properties in the animal’s diet as nutraceuticals, i.e. a feed which combines nutritional value with beneficial effects on animal health [17–20]. Bioactive plants can also be used as potential sources of novel natural products with activity against livestock (and human) parasites [21–23]. Plants or plant components have been used as dewormers for centuries [24], but only in the last 10–15 years has comprehensive scientific research been conducted to evaluate their *in vitro* and *in vivo* activity under experimental and farming conditions [25, 26]. As a result, bioactive forages are now being considered as potential tools for integrated parasite control in livestock [27, 28].

The antiparasitic mechanisms of bioactive forages can be broadly grouped in direct or indirect effects. Direct effects refer to the chemical interaction between plant compounds and specific parasite molecules or structures, resulting in subsequent mortality and removal from the host, whilst indirect effects involve the stimulation or boosting of host responses that can induce worm expulsion, either through overall improved nutrition or a direct interaction between plant compounds and host cells (e.g. leukocytes) [20]. Until now, most of the research focus on antiparasitic forages has been devoted to tannin-rich plants [e.g. sulla (*Hedysarum coronarium*), sainfoin (*Onobrychis viciifolia*), sericea lespedeza (*Lespedeza cuneata*)] [19, 20]. However, a promising non-tanniniferous bioactive forage in temperate regions is chicory (*Cichorium intybus* L., Asteraceae), a multi-purpose crop that has attracted considerable interest from farmers and scientists for its reported anthelmintic effects in livestock. Yet, it is still unclear from the available studies whether chicory diets can exert a broad activity against major parasites in different livestock species, and the levels of chicory in the diet that are required to exert an efficient *in vivo* effect. This knowledge is critical to validate the use of chicory as an antiparasitic forage and an essential step before chicory can appropriately be included in parasite control programs [29]. In addition, the mechanisms behind the reported anthelmintic effects of chicory are still largely unknown and the potential role of its bioactive phytochemicals has not been, until recently, thoroughly investigated.

In this review, we summarise the progress in the study of the antiparasitic activity of chicory and its natural bioactive compounds against GI parasites in livestock, through examination of the published literature. In addition, we identify gaps for future research to advance our understanding on the dietary modulation of parasitism using chicory as a model plant, and perspectives on the practical use of chicory as a nutraceutical forage and a potential source of novel plant compounds with broad antiparasitic activity.

## Chicory: a multi-purpose crop for livestock

Chicory is a perennial, deep-rooting herb that can be found as a wild plant in natural grasslands and roadsides and as cultivated varieties in most temperate areas of the world, including Northern Europe [30, 31]. In some European, Asian and Middle Eastern countries, chicory has traditionally been used for human consumption and as a medicinal plant to treat several diseases, including malaria and digestive, liver and urinary disorders [32, 33]. Based on its current applications, cultivated chicory can be classified in four types [33]: (i) industrial or root chicory used for the production of inulin-type fructans and as coffee substitute; (ii) Brussels or witloof chicory, for production of etiolated leaves (“chicons”); (iii) leaf chicory for human consumption (fresh salad or cooked); and (iv) forage chicory for animal feeding. In the 1980s, selection of chicory for livestock feeding resulted in the release of the first commercial forage variety, “Grasslands Puna” [34]. Since then, several forage chicory cultivars have been developed [35–38]. The use of chicory diets in livestock nutrition has been previously reviewed [37, 39]. In general, forage chicory varieties grow predominantly at temperatures > 10 °C, are rich in minerals (Zn, B, Mn) with variable protein levels [100–250 g crude protein/kg dry matter (DM), depending on N input and defoliation frequency], low neutral detergent fibre levels and high voluntary feed intakes in ruminants [37, 39–41]. Ruminants grazing pure forage chicory have shown similar or higher weight gains in comparison with animals grazed on ryegrass, if provided at comparable levels of DM intake [42–44]. Chicory-based diets have also been reported to sustain milk production in cattle [45–47] and in sheep [48]. However, milk from cows fed pure-chicory had a reportedly bitter taint that has prompted the recommendation to restrict its intake in dairy cows [39].

## Anthelmintic activity of chicory against GI parasites

Over the last two decades, several peer-reviewed studies have explored the *in vivo* effects of dietary forage chicory against GI nematode infections in ruminants. Table 1 summarises the main results reported in these experiments, as well as the ruminant species involved (sheep, cattle and deer), the parasite species (abomasal and/or small intestinal nematodes) and experimental design. If available, information of the chicory cultivar used, the percentage of chicory DM in the diets and the screening of bioactive compounds are also presented. Despite major differences between the studies listed in Table 1, two general conclusions can be drawn. First, feeding with forage chicory can reduce worm faecal egg counts (FEC) and/or worm burdens with abomasal nematodes (*Haemonchus contortus* and *Teladorsagia circumcincta* in sheep and *Ostertagia ostertagi* in cattle), but not infections with intestinal worms in ruminants [49–56]. Secondly, from trials involving experimental infection of worm-naïve animals, feeding levels of ≥ 70% of chicory DM in the diet resulted in a significant reduction of abomasal parasitism with *T. circumcincta* [49, 52] and *O. ostertagi* [56], and in reduction of *H. contortus* FEC [53]. The lowest level of chicory DM in the diet to be associated with an anthelmintic activity was ~50% [54], whereas diets with 24% of chicory DM or lower did not result in any noticeable antiparasitic effect [57, 58].

**Table 1:** Summary of peer-reviewed *in vivo* experiments studying the anthelmintic effects of dietary chicory in ruminants

Livestock species	Chicory DM^a^ (%)	Chicory cv. (sown)	Bioactive compounds in chicory (% DM)^b^	Nematode infection and experimental design^c^	Anthelmintic effects in chicory-fed animals^d^	Reference
Sheep	91	ni (pure)	Total CT = 0.83%	Exp. *T. circumcincta* trickle infection while grazing chicory. After 40 days p.i. animals were stabled, treated with levamisole + ivermectin and challenged with *T. circumcincta* L3	Lower numbers of mid/late L4 and L5/adult worms from the challenge infection. No effect on FEC while grazing on chicory and trickle infected	[61]
	87	ni (pure)	Total CT = 0.05%; total phenolics = 26.2%	Exp. *T. circumcincta* infection	Reduction in adult male worms and lower total adult counts by 43% (ns). No effect on FEC, female per capita fecundity or immature worm counts	[52]
	80	Grasslands Puna (pure)	nr	Exp. *Teladorsagia* spp., *Trichostrongylus* spp., *Oesophagostomum* spp., *Cooperia* spp. and *Nematodirus* spp. infection + reinfection from pasture	Reduced FEC and *Teladorsagia* spp. worm counts	[49]
	80	ni (pure)	Total CT = 0.05%; total phenolics = 26.2%	Exp. *T. colubriformis* infection	No effect on FEC, adult or immature worm counts	[51]
	80	Grasslands Puna (pure)	Total CT = 0.31%	Exp. *H. contortus* and *C. curticei* infection (animals stabled)	Reduced total egg output and FECDM of *H. contortus*. No effect on adult worm counts, on FECDM of *C. curticei* or on total FECDM	[53]
	50	Puna II (pure)	nr	Exp. *T. circumcincta* trickle infection of ewes and reinfection from pasture for ewes and lambs	Reduced FEC in lambs and ewes on chicory and lower *T. circumcincta* L3 recovered from chicory swards (ns)	[54]
	nr	Grasslands Puna (pure)	nr	Natural infection	Reduced abomasal adult counts by 41% and abomasal L4 by 60%. No effect on FEC or intestinal worm counts	[50]
	nr	Grasslands Puna (pure)	Total phenolics = 1.8–2.7%	Natural infections in ewes and their lambs. Ewes were either treated or not treated with anthelmintics	Lower FEC in lambs from untreated ewes on chicory. No effect on FEC of undrenched ewes or on adult worm burden in lambs	[62]
	nr	Oasis (pure)	nr	Natural infection	Reduced FEC. Chicory-fed lambs had lower FAMACHA scores and required less anthelmintic treatments	[55]
	3–6	Grasslands Puna (mixed with RG/WC)	nr	Natural infection	No effect on FEC	[57]
	nr	Grasslands Puna (nr)	nr	Exp. *T. circumcincta* and *T. colubriformis* infection + reinfection from pasture	Reduced FEC and serum pepsinogen levels at Day 42 p.i.	[59]
Cattle	90	Spadona (pure)	Total CT = ni; total SL = 1.7–2.3%	Exp. *O. ostertagi* infections	Reduced worm burdens by 66%. Reduced FEC from Day 21 p.i. onwards	[56]
	70	Spadona (pure)	Total CT = ni; total SL = 1.2%	Exp. *Ostertagia ostertagi* and *Cooperia oncophora* infection (stabled animals)	Reduced *O. ostertagi* worm burdens by 60%. Reduced recovery of *O. ostertagi* L3*.* No effect on total FEC and worm burdens of *C. oncophora*	[56]
	24	Puna II (mixed with RG)	nr	Natural infection	No effect on FEC, proportion of *O. ostertagi* or *Cooperia* spp. L3 in larval cultures, serum pepsinogen or *O. ostertagi*-antibodies	[58]
Deer	56–71	Grasslands Puna (pure)	Total CT = 0.17–0.26%	Natural infection	Fewer clinical signs associated with parasitism, requiring less anthelmintic treatment. No effect on FEC or lungworm L1 counts	[60]

^a^In sward/diet

^b^Detection of bioactive compounds were performed with different methodologies and are not comparable between studies

^c^All studies were grazing experiments, unless otherwise indicated (stabled animals). In all the trials ryegrass or ryegrass/white clover were used as diets for control animals, with the exception of Heckendorn et al. [53] who fed control lambs with ryegrass/lucerne fresh-cut and Miller et al. [55] who used Bermuda grass

^d^In comparison to infected animals fed with control diets

*Abbreviations*: *cv* cultivar, *ns* not significant, *DM* dry matter, *exp*. experimental, *p.i*. post-infection, *CT* condensed tannins, *SL* sesquiterpene lactones, *FEC* faecal egg counts, *FECDM* FEC adjusted per g of faecal DM, *L1* first-stage larvae, *L3* third-stage larvae (free-living), *L4* fourth-stage larvae (immature), *L5* fifth-stage larvae, *nr* not reported, *ni* not identified

Grazing trials in New Zealand were the first to explore the anthelmintic effects of chicory in livestock [49, 59, 60]. Niezen et al. [59] described lower strongyle FEC and serum pepsinogen levels in chicory-fed lambs at Day 42 post-experimental infection with *T. circumcincta* and *Trichostrongylus colubriformis*. Subsequently, Scales et al. [49] reported significantly lower FEC and reduced worm burdens of *Teladorsagia* spp. but not intestinal *Trichostrongylus* spp. in lambs grazing forage chicory, as compared to lambs on control ryegrass (*Lolium perenne*), cooksfoot (*Dactylis glomerata*) or tall fescue (*Festuca arundinacea*). Later studies were in agreement with these findings; e.g. Marley et al. [50] reported a significant reduction of adult and fourth-stage larvae (L4) abomasal nematodes in chicory-fed lambs, while no effect was observed on intestinal worms. Following trials using experimental infections in lambs confirmed that feeding forage chicory-based diets could directly affect the adult burden and FEC of abomasal worms, but not of intestinal nematodes in sheep [51–53]. In cattle, Marley et al. [58] reported that beef steers naturally infected with GI nematodes and grazing a mixed chicory/ryegrass pasture (~24% chicory DM in the field), showed no differences in FEC, *O. ostertagi/Cooperia* spp. third-stage larvae (L3) proportions in faecal cultures, serum pepsinogen or *O. ostertagi-*antibodies levels, when compared to infected controls grazing ryegrass. A recent study described two independent trials exploring the anthelmintic effects of chicory-rich diets (≥ 70% forage chicory in the DM intake) against experimental infections with GI nematodes in cattle [56]. In the first (indoor) experiment, stabled calves infected with *O. ostertagi* and *Cooperia oncophora* and fed chicory silage diets had a largely similar FEC pattern throughout the trial, in comparison with infected control calves fed a balanced protein/energy diet. However, *post-mortem* nematode recovery revealed that calves fed chicory silage had significantly lower worm burdens of *O. ostertagi* (geometric mean reduction of 60%) in comparison with control animals. In contrast, adult counts of *C. oncophora* in the small intestine were not statistically different between groups. In a second (grazing) experiment, calves mono-infected with *O. ostertagi* and grazed without possibilities of reinfections on a pure-chicory field (90% chicory DM in the sward), and later supplemented with chicory silage, had similar FEC in comparison with animals grazing a ryegrass/clover pasture until Day 20 post-infection. However, from Day 22 post-infection onwards a rapid and significant reduction in faecal egg excretion was observed in chicory-fed calves, with a 65% lower mean FEC compared with control animals at the end of the trial. *Post-mortem* worm recovery revealed that calves fed chicory had highly significant reductions in the adult worm burdens of *O. ostertagi* (geometric mean reduction of 66%) in comparison with control animals [56]. These results strongly indicate that chicory feeding can directly reduce abomasal parasitism in ruminants, particularly affecting adult nematodes.

Furthermore, some evidence of potential indirect anthelmintic effects of chicory has been described [61, 62]. Tzamaloukas et al. [61] reported that chicory-fed lambs had higher levels of abomasal mucosal mast cells and globule leucocytes, correlating with lower larval development and worm establishment in these animals. The authors attributed this enhanced cellular immunity to the higher protein content of the chicory diet (crude protein 18.3% in chicory *vs* 11.3% in clover-grass) [61]. Athanasiadou et al. [62] reported lower FEC and higher body weight gains in suckling, naturally-infected lambs from ewes on chicory compared with lambs from ewes grazing grass/clover, although no effect was observed on abomasal/intestinal worm counts in lambs or in the FEC of their periparturient dams. Whereas the reduced FEC in lambs from chicory-fed ewes could be explained by a reduced worm fecundity or reduced challenge from pasture (see below), the authors related the improved performance of these lambs to the higher nutritional quality of chicory rather than to a direct anthelmintic effect, given the similar worm burdens in lambs grazing chicory or grass/clover [62]. These two studies suggest that chicory diets can either indirectly affect GI parasitism by enhancing the immune response [61] or by increasing the animals’ resilience to parasite infections [62]. Further research is needed to confirm these initial observations and to identify if chicory feeding can induce immune-stimulatory effects on concurrent parasitic infections. Moreover, in a different approach, some studies have also suggested that chicory can induce detrimental effects on free-living stages of GI nematodes, potentially due to the activity of undigested bioactive compounds in faeces or due to changes in the sward environment, that could affect nematode survival and the level of parasite challenge from pasture [63–65]. However, at present the strongest evidence exists for a direct anthelmintic effect of chicory inside the host digestive tract, and recent studies combining parasitology and phytochemistry have begun to probe the constituent antiparasitic compounds in chicory.

## Antiparasitic activity of chicory: unveiling the bioactive compounds

Chicory has previously been referred to as a “tanniniferous forage”, although only low levels of condensed tannins (≤ 0.8% in DM) are commonly detected in chicory leaves [52, 53, 56, 66, 67]. As a member of the Asteraceae, chicory is known to synthesise sesquiterpene lactones (SL), a group of biologically-active natural terpenoids which are also partly responsible for its bitter taste [68–72]. Sesquiterpene lactones are present in chicory leaves (~2% in DM; [56]) and roots (~0.4% in DM; [68]) and can be detected as free molecules and/or as glycosides [73]. Beside SL, four other groups of phytochemicals with reported bioactivities have been described in chicory: hydroxycinnamic acids (e.g. chicoric, cholorogenic and caffeic acid derivatives), flavonoids (e.g. quercetin and kaempferol derivatives), anthocyanins and coumarins [68, 73, 74]. The bioactive phytochemicals described in chicory are presented in Table 2, as well as their reported bioactivity. Several of these compounds have been investigated for their bioactivity as single molecules [68, 75–81]. Noticeably, some of the chicory SL have exhibited antimalarial activity against *Plasmodium falciparum* [72], as well as insecticidal [68] and anti-inflammatory [75] properties. Chicory leaves and roots also contain sugars (fructose, glucose and sucrose) and high levels of pectins (uronic acids as building blocks) [82–84]. Chicory roots are rich in inulin-type fructans (comprising up to 70% of the DM in chicory roots), with only traces present in leaves [33, 82, 85]. Inulin is known for its prebiotic properties and pigs supplemented with dried chicory roots (30–35% DM in the diet) had significantly lower FEC and worm burdens of *Oesophagostomum* spp. and reduced larval counts of *Ascaris suum* [86, 87]. These anthelmintic effects are believed to be related to the high content of easily-fermentable fructans (inulin) in the tested chicory roots, which can modify the large intestinal environment and microflora, leading to the increased production of short-chain fatty acids and lactic acids: all factors that can directly or indirectly affect the parasites [88, 89]. Thus, whilst it is probable that chicory roots may have indirect anthelmintic properties in monogastrics due to a prebiotic effect of inulin, the antiparasitic effects of forage chicory observed in grazing ruminants are more likely related to the more abundant bioactive phytochemicals in the leaves.

**Table 2:** Bioactive phytochemicals in chicory (*Cichorium intybus*) and their reported bioactivity

Bioactive compounds	Molecular formula	Reported bioactivity	Model	Reference for bioactivity
Guaianolide sesquiterpene lactones				
Lactucin	C_15_H_16_O_5_	antiprotozoal	*Plasmodium falciparum*	[72]
11,13-dihydrolactucin	C_15_H_18_O_5_	nr		
8-deoxylactucin	C_15_H_16_O_4_	anti-inflammatory	HT29 cells	[75]
		insecticidal	*Schistocerca gregaria*	[68]
11,13-dihydro-8-deoxylactucin	C_15_H_18_O_4_	nr		
Lactucopicrin	C_23_H_22_O_7_	antiprotozoal	*Plasmodium falciparum*	[72]
		insecticidal	*Schistocerca gregaria*	[68]
11,13-dihydrolactucopicrin	C_23_H_24_O_7_	nr		
Hydroxycinnamic acids				
Monocaffeoyl tartaric acid (Caftaric acid)	C_13_H_12_O_9_	nr		
Chlorogenic acid	C_16_H_18_O_9_	antibacterial	*Staphylococcus aureus*, *Streptococcus pneumoniae*, *Bacillus subtilis*, *Escherichia coli*, *Shigella dysenteriae*, *Salmonella typhimurium*	[76]
Caffeic acid	C_9_H_8_O_4_	anticancer	Fibrosarcoma (HT-1080)	[77]
		acaricidal	*Rhipicephalus annulatus*	[78]
Chicoric acid	C_22_H_18_O_12_	insecticidal	*Schistocerca gregaria*	[68]
*p*-Coumaric acid	C_9_H_8_O_3_	anti-inflammatory	Female albino rats of Wistar strain (*in vivo*)	[79]
Caffeoylmalic acid	C_13_H_12_O_8_	nr		
Ferulic acid	C_10_H_10_O_4_	nr		
Flavonoids				
Quercetin 3-O-glucuronide + Luteolin 7-O-glucuronide	C_21_H_18_O_13_	acaricidal	*Rhipicephalus annulatus*	[78]
Quercetin malonyl glucoside	C_24_H_22_O_15_	nr		
Apigenin glucuronide	C_21_H_18_O_11_	nr		
Kaempferol glucuronide	C_21_H_18_O_12_	anticancer and anti-inflammatory	Human pancreatic cancer cells	[80]
Isorhamnetin 3-glucuronide	C_22_H_20_O_13_	nr		
Kaempferol-7-O-(6″-O-malonyl)-glucoside	C_24_H_22_O_14_	nr		
Anthocyanins				
Cyanidin 3-O-glucoside	C_21_H_21_O_11_^+^	nr		
Cyanidin 3-O-(6-malonyl)-glucoside	C_24_H_23_O_14_^+^	anti-inflammatory	Lipid peroxidation and cyclooxygenase (COX-1 and -2) inhibition assay	[81]
Coumarins				
Cichoriin	C_18_H_20_O_3_	insecticidal	*Schistocerca gregaria*	[68]

*Notes*: *p*-Coumaric acid, p-Coumaric acid, Ferulic acid, Isorhamnetin 3-glucuronide and kaempferol-7-O-(6″-O-malonyl)-glucoside were purified and identified by Papetti et al. [74]. Cichorin A was detected and identified by Rees & Harboure [68]. The remaining compounds were identified by Ferioli et al. [73]. All studies reporting bioactivity were performed *in vitro*, unless otherwise indicated (*in vivo*)

*Abbreviations*: *nr* not reported

## Sesquiterpene lactones: the main antiparasitic compounds in chicory?

**Fig. 1:**
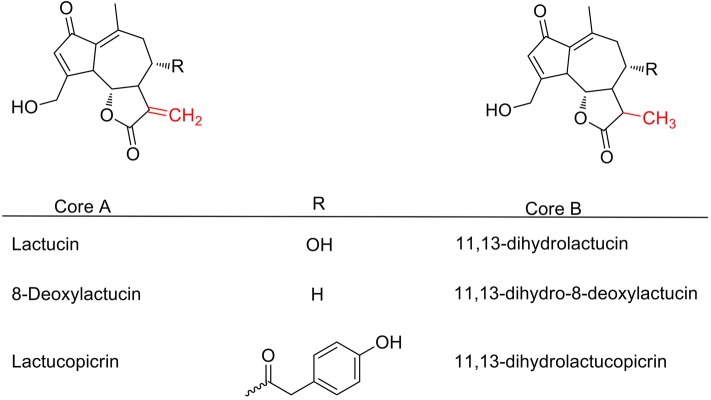
Structures of the guaianolide sesquiterpene lactones reported in chicory

The SL are a group of extremely diverse natural terpenoids, including around 5000 different molecules, which are mainly found in Asteraceae plants [90]. The SL are primarily involved in plant defence against herbivory [68, 91, 92]. Numerous studies have demonstrated substantial biological properties exerted by SL, including antibiotic, antiprotozoal, trematocidal and anticancer activities [91, 93–95]. As an example, artemisinin, a SL originally isolated from the Chinese herb *Artemisia annua* (“qinghao”; “sweet wormwood”) and artemisinin-derivatives (dihydroartemisinin, artesunate, artemether) are currently the front-line antimalarial drugs worldwide [96, 97]. Natural SL are classified in groups depending on their structural conformation, including: germacranolides, eudesmanolides, elemanolides, heliangolides, cadinanolides, eremophilanolides, xanthanolides, guaianolides and pseudoguaianolides, among others [71, 98]. In species of the *Cichorieae* tribe (including chicory) around 360 SL have been reported, all part of the groups of guaianolides (~243 SL), eudesmanolides (~73 SL) or germacranolides (~44 SL) [71]. In chicory leaves, three guaianolide SL and their 11,13-dihydro-derivatives are commonly present: lactucin (LAC), 8-deoxylactucin (8-DOL), lactucopicrin (LCP), 11,13-dihydro-LAC, 11,13-dihydro-8-DOL and 11,13-dihydro-LCP (Table 2, Fig. 1) [68, 70, 99–102]. Other SL have also been detected in chicory rhizomes [103], but not yet described in other parts of the plant.

The anthelmintic potential of chicory SL was first suggested by Schreurs et al. [104] in a study describing the reduced motility of *Dictyocaulus* spp. first-stage larvae (L1) incubated in rumen and abomasal fluid from deer grazing chicory. The authors attributed the observed effects to chicory SL in the digestive fluids, although no detection of SL was reported [104]. Further research by Molan et al. [105] described inhibitory *in vitro* effects of water/ethyl acetate crude SL-extracts from chicory roots on the motility of *Dictyocaulus* spp. L1 and L3 and of GI nematode L3 from deer. However, no chemical identification of SL in the tested chicory extracts was performed [105]. Stronger evidence of the antiparasitic activity of chicory SL has been provided by novel studies involving *in vitro* assays and high-throughput chemical profiling of the tested chicory extracts. Foster et al. [100] described a dose-dependent inhibition of egg hatching in free-living stages of *H. contortus* by SL-rich extracts from two forage chicory cultivars (“Grasslands Puna” and “Forage Feast”). The authors identified the main guaianolide SL of chicory in the tested extracts (LAC, 8-DOL and LCP) and observed an increased ovicidal activity of the Grasslands Puna-extract, which correlated with its higher content of 8-DOL [100]. Recent studies have shown a potent and dose-dependent *in vitro* activity of SL-containing chicory extracts against parasitic stages of cattle nematodes (adult *O. ostertagi* and *C. oncophora*), which are expected to be main targets of dietary SL in the host [102, 106]. The same chicory extracts were subsequently tested *in vitro* against the phylogenetically distinct pig nematodes *A. suum* and *Oesophagostomum dentatum*, observing also a potent activity on these helminths [107]. Chemical profiling of these tested SL-extracts (isolated from two chicory cultivars, “Spadona” and “Puna II”) revealed the presence of LAC, 8-DOL and LCP and their 11,13-dihydro-derivatives, although with differences in the concentration of individual SL between cultivars [102]. Interestingly, these SL-extracts showed distinct anthelmintic potencies against cattle and pig nematodes, and this variable activity was possibly linked to the different SL profiles in the tested extracts [102, 107]. Table 3 summarises the EC_50_ values obtained in *in vitro* assays with parasitic stages of cattle and pig GI nematodes exposed to similar chicory SL-extracts. These results indicate that chicory SL-extracts have potent and broad-spectrum activity against parasitic stages of GI nematodes.

**Table 3:** *In vitro* activity of purified chicory extracts (cv. Spadona) against parasitic stages of gastrointestinal nematodes of livestock

Nematode species	Life-stage	Incubation period with extract (h)	EC_50_ μg/ml	Reference
*Ostertagia ostertagi*	Adults	24	80	[102]
*Cooperia oncophora*	Adults	24	150	[106]
*Ascaris suum*	L3/L4	16/12	81/116	[107]
*Oesophagostomum dentatum*	Adults/L4	24/36	305/372	[107]

*Abbreviation*: EC_50_, effective concentration of purified chicory extracts able to inhibit the motility in 50% of exposed nematodes

**Fig. 2:**
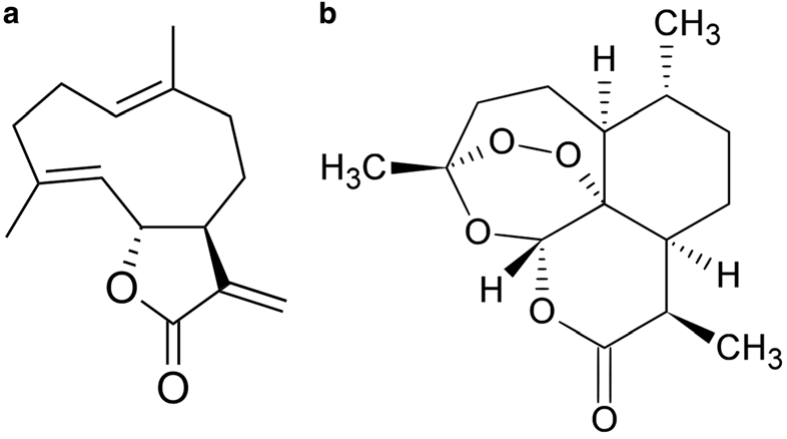
Structures of the sesquiterpene lactones costunolide (**a**) and artemisinin (**b**)

Based on two *in vivo* experiments in which chicory diets (with SL profiling) induced a reduction in adult *O. ostertagi* counts in cattle, the estimated daily consumption of total chicory SL was 27.3 g SL/animal (Exp. 1; 70% chicory DM in diet) and 128.3 g SL/animal (Exp. 2; 90% chicory DM in diet), equivalent to 222 and 761 mg total SL/kg of body weight in animals from Exp. 1 and 2, respectively [56]. However, these estimations do not indicate the concentration of individual SL, which may potentially induce an antiparasitic effect at lower concentrations. Other studies reporting anthelmintic activity of chicory *in vivo* did not describe the content of SL in the diets, although it is expected that the concentrations of total and individual chicory SL vary depending on cultivar, crop age, season of the year and cultivation site [38, 108]. Nevertheless, if SL are truly the compounds behind the direct activity of chicory against GI parasites, these bioactive metabolites should reach the target organs in sufficient concentrations to exert their antiparasitic effect. Potential differences in the activity and concentration of free and glycoside (bound) SL and their metabolisation in different gut compartments can influence their total concentration and activity [100]. Yet, the pharmacokinetics of chicory SL in livestock have not been investigated. Nonetheless, the metabolism of costunolide (Fig. 2a), a germacranolide SL and the parent compound of chicory guaianolide SL [109], has been studied in rats [110–112]. Poor absorption and a peak plasma concentration of 0.024 μg costunolide/ml at 9.0 h post-treatment were observed in rats orally treated with a single dose of costunolide (25 mg/kg) [110]. Similar results were reported in rats orally dosed with a costunolide-containing extract of the herb *Aucklandia lappa* (given at a single dose of 2 g extract/kg, equivalent to 15.7 mg costunolide/kg) [112]. In that study, costunolide was detected in plasma at 5 min after administration and reached a peak concentration of 0.02 μg/ml at 10.5 h post-treatment [112]. These results contrast observations after intravenous injection of costunolide (20 mg/kg), resulting in peak plasma concentrations of 12.3 μg/ml in rats [111]. Previous researchers have explored the pharmacokinetics of other SL such as artemisinin (Fig. 2b) and its derivatives, which are structurally unrelated with chicory guaianolide SL, but knowledge of their metabolism may provide useful insights to elucidate the fate of chicory SL in livestock. Goats treated orally with artemisinin (23 mg/kg) partially absorbed the compound, which was metabolised into the more biologically active dihydroartemisinin (DHA). However, the bioavailability was low (peak of 0.7 μg DHA/mL plasma at 12 h post-treatment) and most of the compound was excreted as unabsorbed artemisinin in faeces (2.4 μg artemisinin/g faeces at 24 h post-treatment) [113]. The same authors incubated artemisinin with alfalfa hay in rumen liquid *in vitro* and did not detect metabolisation of artemisinin to DHA by the rumen microbiota. They also reported a good artemisinin stability at pH 3.0 and 6.8, with a tendency of artemisinin to bind to rumen content and poor solubility in rumen fluid medium [113]. Similarly, Cala et al. [114] reported that sheep treated orally with artemisinin (100 mg/kg) excreted the unabsorbed compound in faeces (peak of 126.5 μg artemisinin/g faeces 24 h post-treatment). These results suggest that oral/dietary SL can survive rumen fermentation and passage through the GI tract, while being partly metabolised and poorly absorbed by ruminants, leading to a low systemic distribution of the bioactive metabolites. However, despite the evidence of a low absorption of oral/dietary SL, the metabolisation of these compounds could lead to the formation of more active compounds inside the digestive tract that can directly affect GI parasites. Previously, it was reported that the guaianolide SL matricin, naturally present in chamomile (*Matricaria recutita*) and other Asteraceae plants, is partly metabolised into the anti-inflammatory compound chamazulene carboxylic acid (a natural profen) in artificial gastric fluid, but not in artificial intestinal fluid [115]. The metabolisation of chicory SL into more active compounds inside the GI tract of livestock warrants further investigation, and this knowledge could help to explain the differences in anthelmintic activities of chicory against abomasal and small intestinal parasites reported in ruminants.

## Elucidating the anthelmintic mechanisms of SL

Guaianolides and other SL are known to exert potent cytotoxic activities, mainly related to the presence of an α-methylene (CH_2_) group attached to the γ-lactone in the SL molecule (Fig. 1: Core A) [91, 116]. This α-methylene group reacts with sulfhydryl (thiol) groups of free cysteine or with cysteine-containing peptides, enzymes or other proteins by a Michael-type addition, leading to an alkylation of cellular macromolecules and disruption of cellular functions (e.g. impairing cell signalling, cell replication and mitochondrial respiration) [98, 116, 117]. As an example, natural SL were reported to reduce the intracellular concentration of free glutathione in *Leishmania mexicana* promastigotes, which led to a toxic intracellular accumulation of reactive oxygen species and blocked cell proliferation *in vitro* [118]. However, there is also evidence that SL may interact with amino acids other than cysteine [91] and SL lacking the α-methylene group (like chicory SL dihydro-derivatives with a CH_3_ group attached to the γ-lactone instead, Fig. 1: Core B) can still exert activity in different biological systems [95, 119], suggesting that there is not only one mechanism of action for all SL. In comparison, artemisinin and its derivatives are thought to have a distinct mechanism of antimalarial activity through the cleavage of a peroxide bridge (present in the artemisinins but absent in chicory SL), which reacts with heme-iron in infected erythrocytes and leads to the production of highly reactive free radicals and parasite death [120].

In previous studies, cattle and swine nematodes exposed to chicory SL-extracts *in vitro* and examined by electron microscopy showed no obvious structural damage in the buccal opening, cuticle or muscle tissue, suggesting that SL may induce anthelmintic activity *via* specific and subtle mechanisms rather than a more generalised degenerative effect [102, 107]. This is in contrast with the reported anthelmintic activities of other plant bioactive compounds such as condensed tannins or cysteine proteinases, which are known to induce marked disruptions in the cuticle of exposed worms [22, 121, 122]. Given the potent and rapid paralysis of nematodes exposed to SL-extracts [102, 107], SL may act as selective inhibitors in the nematode neuromuscular system. A previous study has reported that 8-DOL and LCP isolated from chicory roots can inhibit the activity of acetylcholinesterase (AChE) *in vitro* [123]. A potential inhibition of AChE in nematodes could lead to a build-up of acetylcholine in the neuromuscular junctions resulting in worm paralysis due to permanent contraction [124, 125]. Nematode AChE contains cysteine residues [124], that may interact with the α-CH_2_-γ-lactone group of SL *via* Michael addition, and/or by other alkylation process induced by SL. However, the potential activity of chicory SL on nematode AChE remains to be investigated.

## Further research and perspectives on the use of chicory for parasite control in livestock

One the major gaps in the understanding of the antiparasitic activity of chicory is the precise identification of the responsible compounds. Based on their broad bioactivity, SL are likely the main antiparasitic phytochemicals in chicory, which is supported by previous work reporting differences in the anthelmintic potency of chicory extracts with distinct content of SL [100, 102, 107]. However, further fractionation, compound identification and potential isolation of individual SL, with subsequent bioassays, will be necessary to show conclusively that SL are responsible for the antiparasitic activity of chicory. This research can also help to identify whether individual SL or the combined activity of several SL can induce parasiticidal activity. Notably, it is also necessary to scrutinise if antiparasitic compounds other than SL are present in chicory by studying the activity of different plant fractions and describing their components.

Pharmacokinetic studies are warranted to confirm whether chicory SL and/or other phytochemicals are metabolised into more active compounds in the digestive tract of ruminants and monogastrics, as well as the concentration and activity of these molecules in different organs against various GI parasites. At present, it is unknown whether the discrepant *in vivo* efficacy of chicory against abomasal and intestinal nematodes is explained by distinctly different concentrations of active metabolites reaching the different target organs, as described for condensed tannins in cattle [126]. Recently it has been confirmed that purified chicory extracts can induce dose-dependent and potent effects against adult *C. oncophora in vitro* [106], which suggests that the lack of *in vivo* activity of chicory diets against small intestinal nematodes may be the result of physico-chemical changes in the digestive tract of the host. Therefore, the fate of chicory antiparasitic compounds in the GI tract of ruminants, as well as monogastrics, needs to be investigated. Furthermore, the molecular mechanisms underpinning the direct activity of SL and/or other chicory bioactive compounds against GI parasites need to be elucidated by exploring the potential inhibitory effects on vital processes, like metabolic routes and enzymatic pathways. In addition, it is also pertinent to study the potential parasite adaptation to natural SL and other chicory phytochemicals, as earlier described for condensed tannins and *H. contortus* [127].

The available evidence has confirmed that chicory diets selectively affect abomasal nematodes in infected ruminants, particularly *O. ostertagi* in cattle and *T. circumcincta* in sheep. Only one study has reported *in vivo* effects of chicory against the major abomasal nematode *H. contortus*, observing a significant reduction in FEC but no effect on adult worm burdens in chicory-fed lambs [53]. In relation, a previous study described that feeding of lambs with the SL-containing herb wormwood (*Artemisia absinthium*, Asteraceae) at 20% of DM intake, induced a significant reduction in *H. contortus* worm burdens and FEC [128]. Therefore, additional studies are needed to confirm the activity of chicory towards the highly pathogenic *H. contortus* in small ruminants. Furthermore, all the available *in vivo* studies to date testing the antiparasitic activity of forage chicory diets have been performed in ruminants. Promising *in vitro* activity of SL-extracts of forage chicory against pig nematodes [107] must be confirmed *in vivo*, and potentially also explored in other livestock species.

At present, the antiparasitic activity of chicory has been primarily evaluated in GI helminths and substantial research is needed to explore the potential effects of chicory and its bioactive metabolites against other major GI parasites, such as protozoans. In relation, a previous study confirmed direct antiprotozoal effects of purified SL (LCP and LAC) from chicory roots against the malarial parasite *P. falciparum* (*Honduras*-1 strain) *in vitro*, with total inhibition of parasite growth at 50 and 10 μg/ml of LCP and LAC, respectively [72]. Besides antiplasmodial effects, potent activity of other natural SL against trypanosomes and *Leishmania* spp. has been demonstrated [129–133]. Artemisinin was also reported to induce apoptosis in merozoites of *Eimeria tenella* [134] and inhibition of *Neospora caninum* intracellular multiplication [135]. Therefore, the potential activity of chicory SL against protozoans of veterinary (and medical) importance warrants confirmation. An interesting approach would be to test the activity of chicory and its bioactive compounds against coccidial infections in livestock. Previously, *in vivo* anticoccidial effects of extracts from SL-containing plants (e.g. *A. annua*, *A. absinthium*, *A. sieberi*) and/or single SL (e.g. artemisinin) have been reported in goats [136] and poultry [137–140]. These results strongly support the investigation of the potential anticoccidial activity of chicory in ruminants and monogastrics.

In a nutraceutical approach, the existing evidence sustains the use of chicory-rich diets to target adult abomasal nematode infections in ruminants, which are amongst the most pathogenic and economically important parasitic infections in sheep and cattle [4, 5]. Additional experimental and on-farm studies need to corroborate this selective activity in field conditions and to evaluate the use of chicory as an anthelmintic forage under farming settings. These field studies should primary test the effects of chicory rich-diets on patent infections of abomasal nematodes using FEC with species identification by larval cultures techniques or molecular methods [141, 142], potentially with the use of anti-*Ostertagia* specific antibodies in cattle [143], and whenever possible, confirmed with worm counting post-mortem. The available evidence indicates that levels ≥ 70% chicory DM in the diet can exert anthelmintic effects, but the lower effective intake is currently not established and will probably depend on cultivar, growth conditions, seasonality and other factors that could influence the presence and concentration of bioactive compounds. Notably, the length of the chicory feeding (hours/days) that is required to exert an antiparasitic effect *in vivo* must be elucidated, as previous research has suggested a rapid activity of chicory compounds against parasitic nematodes *in vitro* [102] and a significant and rapid reduction in FEC in chicory-fed calves infected with *O. ostertagi* [56]. Moreover, the consistent differences in anthelmintic potency between chicory cultivars *in vitro*, linked to distinct SL profiles between different varieties [100, 102, 107], remain to be tested *in vivo*. Whether sufficiently high concentrations of chicory can be achieved in commercial farming systems needs additional research, though the use of pure chicory swards for livestock nutrition has been explored [42, 47, 144].

To further validate the practical use of chicory, the economic benefits of including chicory-rich diets in production systems (measured as enhanced animal productivity derived from its antiparasitic effects and improved nutritional value) must be assessed and compared with the extra costs associated with the growth and/or access to chicory. These benefits are expected to vary according to management systems (e.g. availability of land for cultivation of pure chicory, length of the grazing season, parasite exposure, etc.) and in relation to different livestock age groups (e.g. infected with different parasite species). In a nutraceutical approach, and as any other bioactive forage, chicory is also a feed and therefore both its nutritional and antiparasitic value should be evaluated [20]. In addition, several other aspects must be addressed before recommending the use of chicory diets for on-farm parasite control. One issue is the apparent lack of activity against small intestinal worms in ruminants, as grazing animals are normally infected with mixed nematode species. The agronomical shortcoming of cultivating forage chicory should also be considered, like the rapid growth of low-quality reproductive stems in late spring/early summer (from the second year onwards), high vulnerability to damage by grazing stock, its relatively low persistency of around three years and its dormant state during winter, droughts and in dry summers [145, 146]. Another challenge is to ensure chicory feeding for parasitised animals, as the timing of chicory growth and infection dynamics may not match during the grazing season. An alternative could be the preservation of chicory for use when needed, independent of season and pasture availability. Previous studies have explored the conservation of forage chicory as silage to feed cattle and pigs [56, 147, 148] and further research needs to evaluate whether ensiling can affect the concentration and structure of chicory antiparasitic metabolites. However, the ensiling of chicory can be challenging due to the low DM of forage chicory and its broad leaves that can lead to soil contamination during harvest, reducing silage quality [149].

In a future prospect, the comprehensive screening and identification of the main antiparasitic phytochemicals in chicory could help the selection of cultivars with enhanced concentration of these metabolites for use as a nutraceutical forage, or as source of natural parasiticidal compounds. These bioactive metabolites could be selectively isolated to prepare purified extracts that can be used as feed additives for livestock. Such natural antiparasitic feed additives could be given to infected animals in both outdoor (e.g. grazing ruminants) and indoor (e.g. intensive pig) systems. For this purpose, it would be important to define the chicory cultivar(s) and the most suitable part of the plant (leaf, roots) for the extraction of active molecules, as well as the standardisation of the extraction technique to efficiently obtain the desired phytochemicals. In addition, lead parasiticidal molecules identified in chicory could be used to develop novel antiparasitic compounds, as previously discovered with *Artemisia annua* and artemisinin [150]. Once a purified extract or compound(s) can be developed, its best administration route in livestock should be assessed. It is known that SL when given orally to livestock are poorly absorbed and reach a low bioavailability in comparison with injected SL, but these molecules can survive passage through the digestive tract and therefore reach the target GI parasites. Certainly, a better understanding of the pharmacokinetics of chicory bioactive compounds in the host (including the potential metabolisation into more active molecules inside the animal) can help define the best administration routes and delivery strategy to achieve the desired antiparasitic effect. Furthermore, and given the widespread biological activity of SL, it could be expected that these molecules not only interact with parasites in the GI tract, but also with the rumen or intestinal microbiota of the host, which may have a profound influence on animal health and performance. Therefore, a thorough knowledge of the metabolism of SL and their interactions may also shed light on the overall effects of chicory and other SL-rich bioactive forages on animal health and productivity, and to improve our understanding on the interactions between bioactive plant compounds, GI parasites and their hosts.

## Conclusions

Chicory is a bioactive forage with reported antiparasitic activity in ruminants when animals are fed with chicory-rich diets (≥ 70% of chicory DM in the diet). This antiparasitic activity is likely related with its content of bioactive compounds, particularly SL, which have been reported to exert potent effects against helminths and protozoa *in vitro*. The available evidence has confirmed the *in vivo* effects of chicory diets against abomasal nematodes in ruminants, and substantial research is needed to elucidate the potential broad antiparasitic activity of chicory against protozoans and in other livestock species. Additional knowledge of chicory’s antiparasitic metabolites and mechanism(s) of action may indicate ways of improving its efficacy, e.g. by selection of cultivars with increased activity and/or the isolation of leading parasiticides. In addition, future research on chicory may also open new paths for exploring other SL-containing plants and improve our basic understanding of the interactions between bioactive phytochemicals, GI parasites and their hosts.

## Abbreviations

GI: Gastrointestinal
SL: Sesquiterpene lactones
DM: Dry matter
FEC: Faecal egg counts
L1/L3/L4: First/third/fourth-stage larvae
LAC: Lactucin
8-DOL: 8-deoxylactucin
LCP: Lactucopicrin
DHA: Dihydroartemisinin
AChE: Acetylcholinesterase

## References

[1] Fitzpatrick JL (2013). Global food security: the impact of veterinary parasites and parasitologists. Vet Parasitol..

[2] Rist CL, Garchitorena A, Ngonghala CN, Gillespie TR, Bonds MH (2015). The burden of livestock parasites on the poor. Trends Parasitol..

[3] O’Brien D, Scudamore J, Charlier J, Delavergne M. DISCONTOOLS: a database to identify research gaps on vaccines, pharmaceuticals and diagnostics for the control of infectious diseases of animals. BMC Vet Res. 2017;13(1) 10.1186/s12917-016-0931-1 PMC5209808 28049469

[4] Charlier J, van der Voort M, Kenyon F, Skuce P, Vercruysse J (2014). Chasing helminths and their economic impact on farmed ruminants. Trends Parasitol..

[5] Kenyon F, Hutchings F, Morgan-Davies C, van Dijk J, Bartley DJ (2017). Worm control in livestock: bringing science to the field. Trends Parasitol..

[6] Kaplan RM (2004). Drug resistance in nematodes of veterinary importance: a status report. Trends Parasitol..

[7] Waller PJ (2006). Sustainable nematode parasite control strategies for ruminant livestock by grazing management and biological control. Anim Feed Sci Technol..

[8] Woodgate RG, Cornell AJ, Sangster NC, Mayers DL, Sobel JD, Ouellette M, Kaye KS, Marchaim D (2017). Occurrence, measurement and clinical perspectives of drug resistance in important parasitic helminths of livestock. Antimicrobial Drug Resistance.

[9] Waller PJ, Thamsborg SM (2004). Nematode control in ‘green’ ruminant production systems. Trends Parasitol..

[10] Hoste H, Sotiraki S, Mejer H, Heckendorn F, Maurer V, Thamsborg S, Bellon S, Penvern S (2014). Alternatives to synthetic chemical antiparasitic drugs in organic livestock farming in Europe. Organic Farming, Prototype for Sustainable Agricultures.

[11] Romero-González R, Garrido Frenich A, Martínez Vidal JL (2014). Veterinary drugs residues: anthelmintics. Encycl Food Saf..

[12] Rook AJ, Dumont B, Isselstein J, Osoro K, Wallis DeVries MF, Parente G (2004). Matching type of livestock to desired biodiversity outcomes in pastures - a review. Biol Conserv..

[13] Eisler MC, Lee MRF, Tarlton JF, Martin GB, Beddington J, Dungait JAJ (2014). Steps to sustainable livestock. Nature..

[14] Schader C, Muller A, Scialabba NE-H, Hecht J, Isensee A, Erb K-H (2015). Impacts of feeding less food-competing feedstuffs to livestock on global food system sustainability. J R Soc Interface..

[15] Thamsborg SM, Roepstorff A, Larsen M (1999). Integrated and biological control of parasites in organic and conventional production systems. Vet Parasitol..

[16] Thamsborg S, Roepstorff A, Nejsum P, Mejer H (2010). Alternative approaches to control of parasites in livestock: Nordic and Baltic perspectives. Acta Vet Scand..

[17] Githiori JB, Athanasiadou S, Thamsborg SM (2006). Use of plants in novel approaches for control of gastrointestinal helminths in livestock with emphasis on small ruminants. Vet Parasitol..

[18] Athanasiadou S, Githiori J, Kyriazakis I (2007). Medicinal plants for helminth parasite control: facts and fiction. Animal..

[19] Hoste H, Jackson F, Athanasiadou S, Thamsborg SM, Hoskin SO (2006). The effects of tannin-rich plants on parasitic nematodes in ruminants. Trends Parasitol..

[20] Hoste H, Torres-Acosta JFJ, Sandoval-Castro C, Mueller-Harvey I, Sotiraki S, Louvandini H (2015). Tannin containing legumes as a model for nutraceuticals against digestive parasites in livestock. Vet Parasitol..

[21] Kayser O, Kiderlen A, Croft S (2003). Natural products as antiparasitic drugs. Parasitol Res..

[22] Stepek G, Behnke JM, Buttle DJ, Duce IR (2004). Natural plant cysteine proteinases as anthelmintics?. Trends Parasitol..

[23] Behnke JM, Buttle DJ, Stepek G, Lowe A, Duce IR (2008). Developing novel anthelmintics from plant cysteine proteinases. Parasit Vectors..

[24] Waller PJ, Bernes G, Thamsborg SM, Sukura A, Richter SH, Ingebrigtsen K (2001). Plants as de-worming agents of livestock in the Nordic countries: historical perspective, popular beliefs and prospects for the future. Acta Vet Scand..

[25] Athanasiadou S, Kyriazakis I (2007). Plant secondary metabolites: antiparasitic effects and their role in ruminant production systems. Proc Nutr Soc..

[26] Sandoval-Castro CA, Torres-Acosta JFJ, Hoste H, Salem AZM, Chan-Pérez JI (2012). Using plant bioactive materials to control gastrointestinal tract helminths in livestock. Anim Feed Sci Technol..

[27] Charlier J, Thamsborg SM, Bartley DJ, Skuce PJ, Kenyon F, Geurden T (2018). Mind the gaps in research on the control of gastrointestinal nematodes of farmed ruminants and pigs. Transbound Emerg Dis..

[28] Vercruysse J, Charlier J, Van DJ, Morgan ER, Geary T, von Samson-Himmelstjerna G, et al. Control of helminth ruminant infections by 2030. Parasitology. 2018:1–10. 10.1017/S003118201700227X. 10.1017/S003118201700227X 29415781

[29] Houdijk JGM, Kyriazakis I, Kidane A, Athanasiadou S (2012). Manipulating small ruminant parasite epidemiology through the combination of nutritional strategies. Vet Parasitol..

[30] Høgh-Jensen H, Nielsen B, Thamsborg SM (2006). Productivity and quality, competition and facilitation of chicory in ryegrass/legume-based pastures under various nitrogen supply levels. Eur J Agron..

[31] Lucchin M, Varotto S, Barcaccia G, Chicory PP, Prohens-Tomás J, Nuez F (2008). Endive. Vegetables I: Asteraceae, Brassicaceae, Chenopodicaceae, and Cucurbitaceae.

[32] Hitova A, Melzig M (2014). *Cichorium intybus* L. Zeitschrift für Phyther..

[33] Street RA, Sidana J, Prinsloo G. *Cichorium intybus*: Traditional uses, phytochemistry, pharmacology, and toxicology. Evid Based Complement Alternat Med. 2013; 10.1155/2013/579319. 10.1155/2013/579319 PMC3860133 24379887

[34] Rumball W (1986). Grasslands Puna’ chicory (*Cichorium intybus* L.). New Zeal J Exp Agric..

[35] Rumball W, Keogh R, Miller J, Claydon R (2003). ‘Choice’ forage chicory (*Cichorium intybus* L .). New Zeal J Agric Res..

[36] Rumball W, Skipp R, Keogh R, Claydon R (2003). ‘Puna II’ forage chicory (*Cichorium intybus* L.). New Zeal J Agric Res..

[37] Li G, Kemp P (2005). Forage chicory (*Cichorium intybus* L.): a review of its agronomy and animal production. Adv Agron..

[38] Foster J, Clapham WM, Belesky DP, Labreveux M, Hall MH, Sanderson MA (2006). Influence of cultivation site on sesquiterpene lactone composition of forage chicory (*Cichorium intybus* L.). J Agric Food Chem..

[39] Barry T (1998). The feeding value of chicory (*Cichorium intybus*) for ruminant livestock. J Agric Sci..

[40] Belesky DP, Turner KE, Fedders JM, Ruckle JM (2001). Mineral composition of swards containing forage chicory. Agron J..

[41] Fraser T, Cosgrove G, Thomas W, Stevens D, Hickey M (1988). Performance of grasslands puna chicory. Proc New Zeal Grassl Assoc..

[42] Clark D, Anderson C, Hongwen G (1990). Liveweight gain and intake of Friesian bulls grazing ‘Grasslands Puna’ chicory (*Cichorium intybus* L.) or pasture. New Zeal J Agric Res..

[43] Hoskin SO, Barry TN, Wilson PR, Charleston WAG, Kemp PD (1999). Growth and carcass production of young farmed deer grazing sulla (*Hedysarum coronarium*), chicory (*Cichorium intybus*), or perennial ryegrass (*Lolium perenne*) white clover (*Trifolium repens*) pasture in New Zealand. New Zeal J Agric Res..

[44] Kidane A, Sørheim K, Eik LO, Steinshamn H (2014). Growth and chemical composition of chicory and performance of lambs grazing chicory relative to grass-clover mixtures. Acta Agr Scand A-An..

[45] Chapman D, Tharmaraj J, Nie Z (2008). Milk-production potential of different sward types in a temperate southern Australian environment. Grass Forage Sci..

[46] Woodward SL, Waugh CD, Roach CG, Fynn D, Phillips J (2013). Are diverse species mixtures better pastures for dairy farming?. Proc New Zeal Grassl Assoc..

[47] Muir S, Ward G, Jacobs J (2014). Milk production and composition of mid-lactation cows consuming perennial ryegrass-and chicory-based diets. J Dairy Sci..

[48] Di Grigoli A, Todaro M, Di Miceli G, Genna V, Tornambè G, Alicata ML (2012). Effects of continuous and rotational grazing of different forage species on ewe milk production. Small Rumin Res..

[49] Scales GH, Knight TL, Saville DJ (1995). Effect of herbage species and feeding level on internal parasites and production performance of grazing lambs. New Zeal J Agric Res..

[50] Marley CL, Cook R, Keatinge R, Barrett J, Lampkin N (2003). The effect of birdsfoot trefoil (*Lotus corniculatus*) and chicory (*Cichorium intybus*) on parasite intensities and performance of lambs naturally infected with helminth parasites. Vet Parasitol..

[51] Athanasiadou S, Tzamaloukas O, Kyriazakis I, Jackson F, Coop RL (2005). Testing for direct anthelmintic effects of bioactive forages against *Trichostrongylus colubriformis* in grazing sheep. Vet Parasitol..

[52] Tzamaloukas O, Athanasiadou S, Kyriazakis I, Jackson F, Coop RL (2005). The consequences of short-term grazing of bioactive forages on established adult and incoming larvae populations of *Teladorsagia circumcincta* in lambs. Int J Parasitol..

[53] Heckendorn F, Häring DA, Maurer V, Senn M, Hertzberg H (2007). Individual administration of three tanniferous forage plants to lambs artificially infected with *Haemonchus contortus* and *Cooperia curticei*. Vet Parasitol..

[54] Kidane A, Houdijk JGM, Athanasiadou S, Tolkamp BJ, Kyriazakis I (2010). Effects of maternal protein nutrition and subsequent grazing on chicory (*Cichorium intybus*) on parasitism and performance of lambs. J Anim Sci..

[55] Miller MC, Duckett SK, Andrae JG (2011). The effect of forage species on performance and gastrointestinal nematode infection in lambs. Small Rumin Res..

[56] Peña-Espinoza M, Thamsborg SM, Desrues O, Hansen TVA, Enemark HL (2016). Anthelmintic effects of forage chicory (*Cichorium intybus*) against gastrointestinal nematode parasites in experimentally infected cattle. Parasitology..

[57] Nielsen BK, Thamsborg SM, Hansen H, Ranvig H, Høgh-Jensen H (2009). Effects of including chicory in perennial ryegrass-white clover leys on production and health in organic lambs. Livest Sci..

[58] Marley CL, Fychan R, Davies J, Scollan N, Richardson R, Theobald V (2014). Effects of chicory/perennial ryegrass swards compared with perennial ryegrass swards on the performance and carcass quality of grazing beef steers. PLoS One.

[59] Niezen J, Waghorn TS, Raufaut K, Robertson HA, McFarlane RG (1994). Lamb weight gain and faecal egg count when grazing one of seven herbages and dosed with larvae for six weeks. Proc New Zeal Soc Anim Prod.

[60] Hoskin SO, Barry TN, Wilson PR, Charleston WAG, Hodgson J (1999). Effects of reducing anthelmintic input upon growth and faecal egg and larval counts in young farmed deer grazing chicory (*Cichorium intybus*) and perennial ryegrass (*Lolium perenne*)/white clover (*Trifolium repens*) pasture. J Agric Sci..

[61] Tzamaloukas O, Athanasiadou S, Kyriazakis I, Huntley JF, Jackson F (2006). The effect of chicory (*Cichorium intybus*) and sulla (*Hedysarum coronarium*) on larval development and mucosal cell responses of growing lambs challenged with *Teladorsagia circumcincta*. Parasitology..

[62] Athanasiadou S, Gray D, Younie D, Tzamaloukas O, Jackson F, Kyriazakis I (2007). The use of chicory for parasite control in organic ewes and their lambs. Parasitology..

[63] Moss RA, Vlassoff A (1993). Effect of herbage species on gastro-intestinal roundworm populations and their distribution. New Zeal J Agric Res..

[64] Niezen JH, Charleston WAG, Hodgson J, Miller CM, Waghorn TS, Robertson HA (1998). Effect of plant species on the larvae of gastrointestinal nematodes which parasitise sheep. Int J Parasitol..

[65] Marley CL, Cook R, Barrett J, Keatinge R, Lampkin NH (2006). The effects of birdsfoot trefoil (*Lotus corniculatus*) and chicory (*Cichorium intybus*) when compared with perennial ryegrass (*Lolium perenne*) on ovine gastrointestinal parasite development, survival and migration. Vet Parasitol..

[66] Jackson FS, McNabb WC, Barry TN, Foo YL, Peters JS (1996). The condensed tannin content of a range of subtropical and temperate forages and the reactivity of condensed tannin with ribulose-1, 5-bis-phosphate carboxylase (Rubisco) protein. J Sci Food Agric..

[67] Lombardi D, Vasseur E, Berthiaume R, DeVries TJ, Bergeron R (2015). Feeding preferences and voluntary feed intake of dairy cows: effect of conservation and harvest time of birdsfoot trefoil and chicory. J Dairy Sci..

[68] Rees S, Harborne J (1985). The role of sesquiterpene lactones and phenolics in the chemical defence of the chicory plant. Phytochemistry..

[69] Peters AM, van Amerongen A (1997). A study on the effects of sample pre-treatment on the amount of sesquiterpene lactones found in chicory (*Cichorium intybus* L.) by ELISA and by HPLC. Zeitschrift für Leb und Forsch..

[70] Kisiel W, Zielin K (2001). Guaianolides from *Cichorium intybus* and structure revision of *Cichorium* sesquiterpene lactones. Phytochemistry..

[71] Zidorn C (2008). Sesquiterpene lactones and their precursors as chemosystematic markers in the tribe Cichorieae of the Asteraceae. Phytochemistry..

[72] Bischoff TA, Kelley CJ, Karchesy Y, Laurantos M, Nguyen-Dinh P, Arefi AG (2004). Antimalarial activity of lactucin and lactucopicrin: sesquiterpene lactones isolated from *Cichorium intybus* L. J Ethnopharmacol..

[73] Ferioli F, Manco M, D’Antuono L (2015). Variation of sesquiterpene lactone and phenolic content in chicory and endive germplasm. J Food Compos Anal..

[74] Papetti A, Maietta M, Corana F, Marrubini G, Gazzani G (2017). Polyphenolic profile of green/red spotted Italian *Cichorium intybus* salads by RP-HPLC-PDA-ESI-MSn. J Food Compos Anal..

[75] Cavin C, Delannoy M, Malnoe A, Debefve E, Touché A, Courtois D (2005). Inhibition of the expression and activity of cyclooxygenase-2 by chicory extract. Biochem Biophys Res Commun..

[76] Lou Z, Wang H, Zhu S, Ma C, Wang Z (2011). Antibacterial activity and mechanism of action of chlorogenic acid. J Food Sci..

[77] Prasad N, Karthikeyan A, Karthikeyan S, Reddy B (2011). Inhibitory effect of caffeic acid on cancer cell proliferation by oxidative mechanism in human HT-1080 fibrosarcoma cell line. Mol Cell Biochem..

[78] Ravindran R, Chithra ND, Deepa PE, Ajithkumar KG, Chandrasekhar L, Sreelekha K, et al. *In vitro* effects of caffeic acid, nortriptyline, precocene I and quercetin against *Rhipicephalus annulatus* (Acari: Ixodidae). Exp Appl Acarol. 2017;71:183–193. 10.1007/s10493-017-0105-2 28110429

[79] Pragasam SJ, Venkatesan V, Rasool M (2013). Immunomodulatory and anti-inflammatory effect of p-coumaric acid, a common dietary polyphenol on experimental inflammation in rats. Inflammation..

[80] Chen AY, Chen YC (2013). A review of the dietary flavonoid, kaempferol on human health and cancer chemoprevention. Food Chem..

[81] Mulabagal V, Wang H, Ngouajio M, Nair MG (2009). Characterization and quantification of health beneficial anthocyanins in leaf chicory (*Cichorium intybus*) varieties. Eur Food Res Technol..

[82] Ernst M, Chatterton NJ, Harrison PA (1995). Carbohydrate changes in chicory (*Cichorium intybus* L. var. foliosum) during growth and storage. Sci Hortic..

[83] Ivarsson E, Liu HY, Dicksved J, Roos S, Lindberg JE (2012). Impact of chicory inclusion in a cereal-based diet on digestibility, organ size and faecal microbiota in growing pigs. Animal..

[84] Liu H, Ivarsson E, Lundh T, Lindberg JE (2013). Chicory (*Cichorium intybus* L.) and cereals differently affect gut development in broiler chickens and young pigs. J Anim Sci Biotechnol..

[85] Roberfroid MB (2005). Introducing inulin-type fructans. Br J Nutr..

[86] Mejer H, Roepstorff A, Thamsborg SM (2009). The effect of dietary inclusion of dried chicory roots on *Oesophagostomum* spp. infections in naturally infected sows. 22nd International Conference of the World Association for the Advancement of Veterinary Parasitology.

[87] Jensen A, Mejer H, Mølbak L, Langkjær M, Jensen TK, Angen Ø (2011). The effect of a diet with fructan-rich chicory roots on intestinal helminths and microbiota with special focus on *Bifidobacteria* and *Campylobacter* in piglets around weaning. Animal..

[88] Petkevičius S, Knudsen KEB, Murrell KD, Wachmann H (2003). The effect of inulin and sugar beet fibre on *Oesophagostomum dentatum* infection in pigs. Parasitology..

[89] Petkevičius S, Murrell KD, Knudsen KEB, Jørgensen H, Roepstorff A, Laue A (2004). Effects of short-chain fatty acids and lactic acids on survival of *Oesophagostomum dentatum* in pigs. Vet Parasitol..

[90] Seaman FC (1982). Sesquiterpene lactones as taxonomic characters in the Asteraceae. Bot Rev..

[91] Schmidt TJ (2006). Structure-activity relationships of sesquiterpene lactones. Stud Nat Prod Chem..

[92] Gershenzon J, Dudareva N (2007). The function of terpene natural products in the natural world. Nat Chem Biol..

[93] Chadwick M, Trewin H, Gawthrop F, Wagstaff C (2013). Sesquiterpenoids lactones: benefits to plants and people. Int J Mol Sci..

[94] Keiser J, Utzinger J (2007). Food-borne trematodiasis: current chemotherapy and advances with artemisinins and synthetic trioxolanes. Trends Parasitol..

[95] Ghantous A, Gali-Muhtasib H, Vuorela H, Saliba N, Darwiche N (2010). What made sesquiterpene lactones reach cancer clinical trials?. Drug Discov Today..

[96] Meshnick SR (2002). Artemisinin: mechanisms of action, resistance and toxicity. Int J Parasitol..

[97] Tilley L, Straimer J, Gnädig NF, Ralph SA, Fidock DA (2016). Artemisinin action and resistance in *Plasmodium falciparum*. Trends Parasitol..

[98] Amorim MHR, Gil da Costa RM, Lopes C, Bastos MMSM (2013). Sesquiterpene lactones: adverse health effects and toxicity mechanisms. Crit Rev Toxicol..

[99] Price K, DuPont M, Shepherd R, Chan H, Fenwick G (1990). Relationship between the chemical and sensory properties of exotic salad crops–coloured lettuce (*Lactuca sativa*) and chicory (*Cichorium intybus*). J Sci Food Agric..

[100] Foster J, Cassida K, Turner K. *In vitro* analysis of the anthelmintic activity of forage chicory (*Cichorium intybus* L.) sesquiterpene lactones against a predominantly *Haemonchus contortus* egg population. Vet Parasitol. 2011;180:298–306. 10.1016/j.vetpar.2011.03.013 21477927

[101] Ferioli F, D’Antuono LF (2012). An update procedure for an effective and simultaneous extraction of sesquiterpene lactones and phenolics from chicory. Food Chem..

[102] Peña-Espinoza M, Boas U, Williams AR, Thamsborg SM, Simonsen HT, Enemark HL. Sesquiterpene lactone containing extracts from two cultivars of forage chicory (*Cichorium intybus*) show distinctive chemical profiles and *in vitro* activity against *Ostertagia ostertagi*. Int J Parasitol Drugs Drug Resist. 2015;5:191–200. 10.1016/j.ijpddr.2015.10.002 PMC4847107 27120066

[103] Nishimura H, Kondo Y, Nagasaka T, Satoh A (2000). Allelochemicals in chicory and utilization in processed foods. J Chem Ecol..

[104] Schreurs NM, Molan AL, Barry N, Mcnabb WC. Effects of grazing undrenched weaner deer on chicory or perennial ryegrass/white clover pasture on the viability of gastrointestinal nematodes and lungworms. Vet Rec. 2002:348–53. 10.1136/vr.151.12.348 12371691

[105] Molan AL, Duncan AJ, Barry TN, McNabb WC (2003). Effects of condensed tannins and crude sesquiterpene lactones extracted from chicory on the motility of larvae of deer lungworm and gastrointestinal nematodes. Parasitol Int..

[106] Peña-Espinoza M, Williams AR, Thamsborg SM, Simonsen HT, Enemark HL (2017). Anthelmintic effects of forage chicory (*Cichorium intybus*) against free-living and parasitic stages of *Cooperia oncophora*. Vet Parasitol..

[107] Williams AR, Peña-Espinoza M, Boas U, Simonsen HT, Enemark HL, Thamsborg SM. Anthelmintic activity of chicory (*Cichorium intybus*): *in vitro* effects on swine nematodes and relationship to sesquiterpene lactone composition. Parasitology. 2016;143:770–7. 10.1017/S0031182016000287 26935644

[108] Foster J, Cassida K, Sanderson M (2011). Seasonal variation in sesquiterpene lactone concentration and composition of forage chicory (*Cichorium intybus* L.) cultivars. Grass Forage Sci..

[109] de Kraker JW, Franssen MCR, Joerink M, De Groot A, Bouwmeester HJ (2002). Biosynthesis of costunolide, dihydrocostunolide, and leucodin. Demonstration of cytochrome P450-catalyzed formation of the lactone ring present in sesquiterpene lactones of chicory. Plant Physiol..

[110] Hu F, Feng S, Wu Y, Bi Y, Wang C, Li W (2011). Quantitative analysis of costunolide and dehydrocostuslactone in rat plasma by ultraperformance liquid chromatography-electrospray ionization-mass spectrometry. Biomed Chromatogr..

[111] Peng Z, Wang Y, Gu X, Guo X, Yan C (2014). Study on the pharmacokinetics and metabolism of costunolide and dehydrocostus lactone in rats by HPLC-UV and UPLC-Q-TOF/MS. Biomed Chromatogr..

[112] Zhang J, Hu X, Gao W, Qu Z, Guo H, Liu Z, et al. Pharmacokinetic study on costunolide and dehydrocostuslactone after oral administration of traditional medicine Aucklandia lappa Decne by LC/MS/MS. J Ethnopharmacol. 2014;151:191–7. 10.1016/j.jep.2013.10.024 24216164

[113] Ferreira JFS, Gonzalez JM (2008). Chemical and biological stability of artemisinin in bovine rumen fluid and its kinetics in goats (*Capra hircus*). Brazil J Vet Parasitol..

[114] Cala AC, Ferreira JFS, Chagas ACS, Gonzalez JM, Rodrigues RAF, Foglio MA, et al. Anthelmintic activity of *Artemisia annua* L. extracts *in vitro* and the effect of an aqueous extract and artemisinin in sheep naturally infected with gastrointestinal nematodes. Parasitol Res. 2014;113:2345–53. 10.1007/s00436-014-3891-z 24802864

[115] Ramadan M, Goeters S, Watzer B, Krause E, Lohmann K, Bauer R (2006). Chamazulene carboxylic acid and matricin: a natural profen and its natural prodrug, identified through similarity to synthetic drug substances. J Nat Prod..

[116] Simonsen H, Weitzel C, Christensen S. Guaianolide sesquiterpenoids: pharmacology and biosynthesis. In: Ramawat K, Mérillon JM, editors. Natural Products. Berlin-Heidelberg: Springer; 2013. p. 3069–98.

[117] Wagner S, Kratz F, Merfort I. *In vitro* behaviour of sesquiterpene lactones and sesquiterpene lactone-containing plant preparations in human blood, plasma and human serum albumin solutions. Planta Med. 2004;70:227–33. 10.1055/s-2004-815539 15114499

[118] Barrera P, Sülsen VP, Lozano E, Rivera M, Beer MF, Tonn C, et al. Natural sesquiterpene lactones induce oxidative stress in *Leishmania mexicana*. Evid Based Complement Alternat Med. 2013;2013:163404. 10.1155/2013/163404 PMC3687511 23861697

[119] Lee K, Ibuka T, Rong-Yang W, Geissman T (1977). Structure-antimicrobial activity relationships among the sesquiterpene lactones and related compounds. Phytochemistry..

[120] Ridley RG (2003). Malaria: to kill a parasite. Nature..

[121] Brunet S, Fourquaux I, Hoste H (2011). Ultrastructural changes in the third-stage, infective larvae of ruminant nematodes treated with sainfoin (*Onobrychis viciifolia*) extract. Parasitol Int..

[122] Martínez-Ortíz-de-Montellano C, Arroyo-López C, Fourquaux I, JFJ T-A, Sandoval-Castro C, Hoste H. Scanning electron microscopy of *Haemonchus contortus* exposed to tannin-rich plants under *in vivo* and *in vitro* conditions. Exp Parasitol. 2013;133:281–6. 10.1016/j.exppara.2012.11.024 23246590

[123] Rollinger JM, Mock P, Zidorn C, Ellmerer EP, Langer T, Stuppner H (2005). Application of the in combo screening approach for the discovery of non-alkaloid acetylcholinesterase inhibitors from *Cichorium intybus*. Curr Drug Discov Technol..

[124] Selkirk ME, Lazari O, Matthews JB (2005). Functional genomics of nematode acetylcholinesterases. Parasitology..

[125] Martin RJ (1997). Modes of action of anthelmintic drugs. Vet J..

[126] Desrues O, Mueller-Harvey I, Pellikaan WF, Enemark HL, Thamsborg SM (2017). Condensed tannins in the gastrointestinal tract of cattle after sainfoin (*Onobrychis viciifolia*) intake and their possible relationship with anthelmintic effects. J Agric Food Chem..

[127] Calderón-Quintal J, Torres- Acosta J, Sandoval-Castro C, Alonso M, Hoste H, Aguilar-Caballero A (2010). Adaptation of *Haemonchus contortus* to condensed tannins: can it be possible?. Arch Med Vet..

[128] Valderrábano J, Calvete C, Uriarte J (2010). Effect of feeding bioactive forages on infection and subsequent development of *Haemonchus contortus* in lamb faeces. Vet Parasitol..

[129] de Toledo JS, Ambrósio SR, Borges CHG, Manfrim V, Cerri DG, Cruz AK, et al. *In vitro* leishmanicidal activities of sesquiterpene lactones from *Tithonia diversifolia* against *Leishmania braziliensis* promastigotes and amastigotes. Molecules. 2014;19:6070–9. 10.3390/molecules19056070 PMC6271005 24830711

[130] Zimmermann S, Fouché G, De Mieri M, Yoshimoto Y, Usuki T, Nthambeleni R (2014). Structure-activity relationship study of sesquiterpene lactones and their semi-synthetic amino derivatives as potential antitrypanosomal products. Molecules..

[131] Schmidt TJ, Nour AMM, Khalid SA, Kaiser M, Brun R (2009). Quantitative structure - antiprotozoal activity relationships of sesquiterpene lactones. Molecules..

[132] Ibrahim M, Aliyu A, Abdullahi H, Solomon T, Toko E, Garba A (2013). Lactone-rich fraction from *Vernonia blumeoides*: antitrypanosomal activity and alleviation of the parasite-induced anemia and organ damage. J Nat Med..

[133] Sülsen VP, Frank FM, Cazorla SI, Anesini CA, Malchiodi EL, Freixa B (2008). Trypanocidal and leishmanicidal activities of sesquiterpene lactones from *Ambrosia tenuifolia* Sprengel (Asteraceae). Antimicrob Agents Chemother..

[134] Mo P, Ma Q, Zhao X, Cheng N, Tao J, Li J (2014). Apoptotic effects of antimalarial artemisinin on the second generation merozoites of *Eimeria tenella* and parasitized host cells. Vet Parasitol..

[135] Kim JT, Park JY, Seo HS, Oh HG, Noh JW, Kim JH, et al. *In vitro* antiprotozoal effects of artemisinin on *Neospora caninum*. Vet Parasitol. 2002;103:53–63. 10.1016/s0304-4017(01)00580-5 11751000

[136] Iqbal A, Tariq KA, Wazir VS, Singh R (2013). Antiparasitic efficacy of *Artemisia absinthium*, toltrazuril and amprolium against intestinal coccidiosis in goats. J Parasit Dis..

[137] De AGF, Horsted K, Thamsborg SM, Kyvsgaard NC, Ferreira JFS, Hermansen JE (2012). Use of *Artemisia annua* as a natural coccidiostat in free-range broilers and its effects on infection dynamics and performance. Vet Parasitol..

[138] Kaboutari J, Arab HA, Ebrahimi K, Rahbari S (2014). Prophylactic and therapeutic effects of a novel granulated formulation of *Artemisia* extract on broiler coccidiosis. Trop Anim Health Prod..

[139] Drǎgan L, Györke A, Ferreira JFS, Pop IA, Dunca I, Drǎgan M (2014). Effects of *Artemisia annua* and *Foeniculum vulgare* on chickens highly infected with *Eimeria tenella* (phylum Apicomplexa). Acta Vet Scand..

[140] Pop L, Györke A, Tăbăran AF, Dumitrache MO, Kalmár Z, Magdaş C (2015). Effects of artemisinin in broiler chickens challenged with *Eimeria acervulina*, *E. maxima* and *E. tenella* in battery trials. Vet Parasitol..

[141] Höglund J, Engström A, von Samson-Himmelstjerna G, Demeler J, Tydén E (2013). Real-time PCR detection for quantification of infection levels with *Ostertagia ostertagi* and *Cooperia oncophora* in cattle faeces. Vet Parasitol..

[142] Roeber F, Morrison A, Casaert S, Smith L, Claerebout E, Skuce P (2017). Multiplexed-tandem PCR for the specific diagnosis of gastrointestinal nematode infections in sheep: an European validation study. Parasit Vectors..

[143] Charlier J, Duchateau L, Claerebout E, Vercruysse J (2005). Assessment of the repeatability of a milk *Ostertagia ostertagi* ELISA and effects of sample preparation. Prev Vet Med..

[144] Parish JA, Parish JR (2012). Best TF, Saunders JR. Comparison of chicory and annual ryegrass for spring stockering of beef steers. Prof Anim Sci..

[145] Sanderson MA, Labreveux M, Hall MH, Elwinger GF (2003). Nutritive value of chicory and English plantain forage. Crop Sci..

[146] Lee JM, Hemmingson NR, Minnee EMK, Clark CE (2015). Management strategies for chicory (*Cichorium intybus*) and plantain (*Plantago lanceolata*): impact on dry matter yield, nutritive characteristics and plant density. Crop Pasture Sci..

[147] Kälber T, Kreuzer M, Leiber F (2013). Effect of feeding buckwheat and chicory silages on fatty acid profile and cheese-making properties of milk from dairy cows. J Dairy Res..

[148] Åkerfeldt MP, Nihlstrand J, Neil M. Chicory and red clover silage in diets to finishing pigs - influence on performance, time budgets and social interactions. Org Agric. 2018. 10.1007/s13165-018-0216-z.

[149] Kälber T, Kreuzer M, Leiber F (2012). Silages containing buckwheat and chicory: quality, digestibility and nitrogen utilisation by lactating cows. Arch Anim Nutr..

[150] Wink M (2012). Medicinal plants: A source of anti-parasitic secondary metabolites. Molecules..

